# Supplementary material for: Genomic programming of IRF4-expressing human Langerhans cells
Source: Nat Commun. 2020 Jan 16;11:313. doi: 10.1038/s41467-019-14125-x (PMC6965086; doi:10.1038/s41467-019-14125-x)
Supplement: Supplementary file 1 — Supplementary Information [file 41467_2019_14125_MOESM1_ESM.pdf]

Genomic programming of IRF4-expressing human Langerhans cells

Sirvent S. *et al*

Supplementary Information

Supplementary Figure 1. System for analysing human LCs and control of antigen cross-presentation

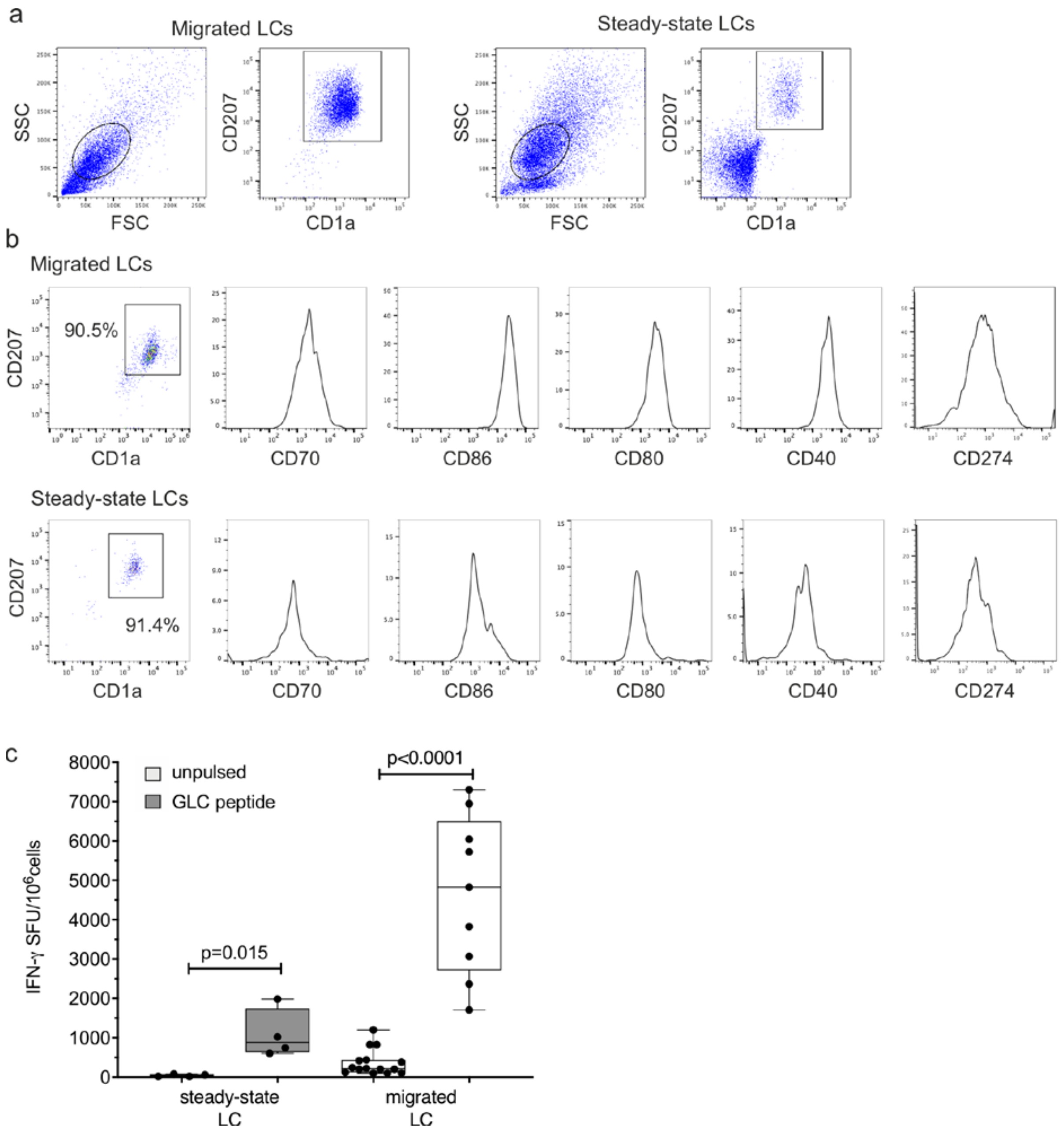

a) Gating strategy for identification of human migrated and steady state Langerhans cells
b) Flow cytometry assessment of steady-state and migrated LCs. LCs were enumerated as CD207/CD1a/HLA-DR high cells. Expression of co-stimulatory molecules critical for CD8 T cell activation during antigen presentation was measured. A representative example of n>5 independent donors.

c) IFN-γ secretion by EBV-specific CD8 T cell line stimulated with antigen presenting LCs in the context of MHC I HLA-A2. Steady-state or migrated LCs were pulsed with 9 amino acid peptide GLC, an EBV epitope (GLC, dark grey), or unpulsed (light grey). Pulsed or unpulsed (light gray) LCs were stimulated with TNF (24h) and then assayed for IFN-γ secretion. ELISpot assay, n=4-6 independent experiments, paired t-test, box and whiskers show min and max value, line at median. Source data are provided as a Source Data file

Supplementary Figure 2 Transcriptional programming of migrated human LCs

a

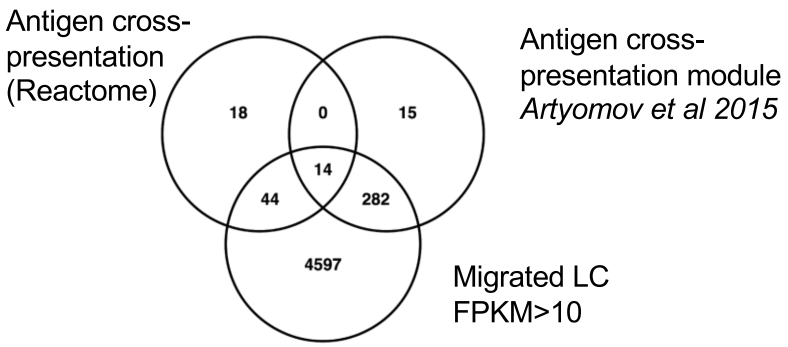

b

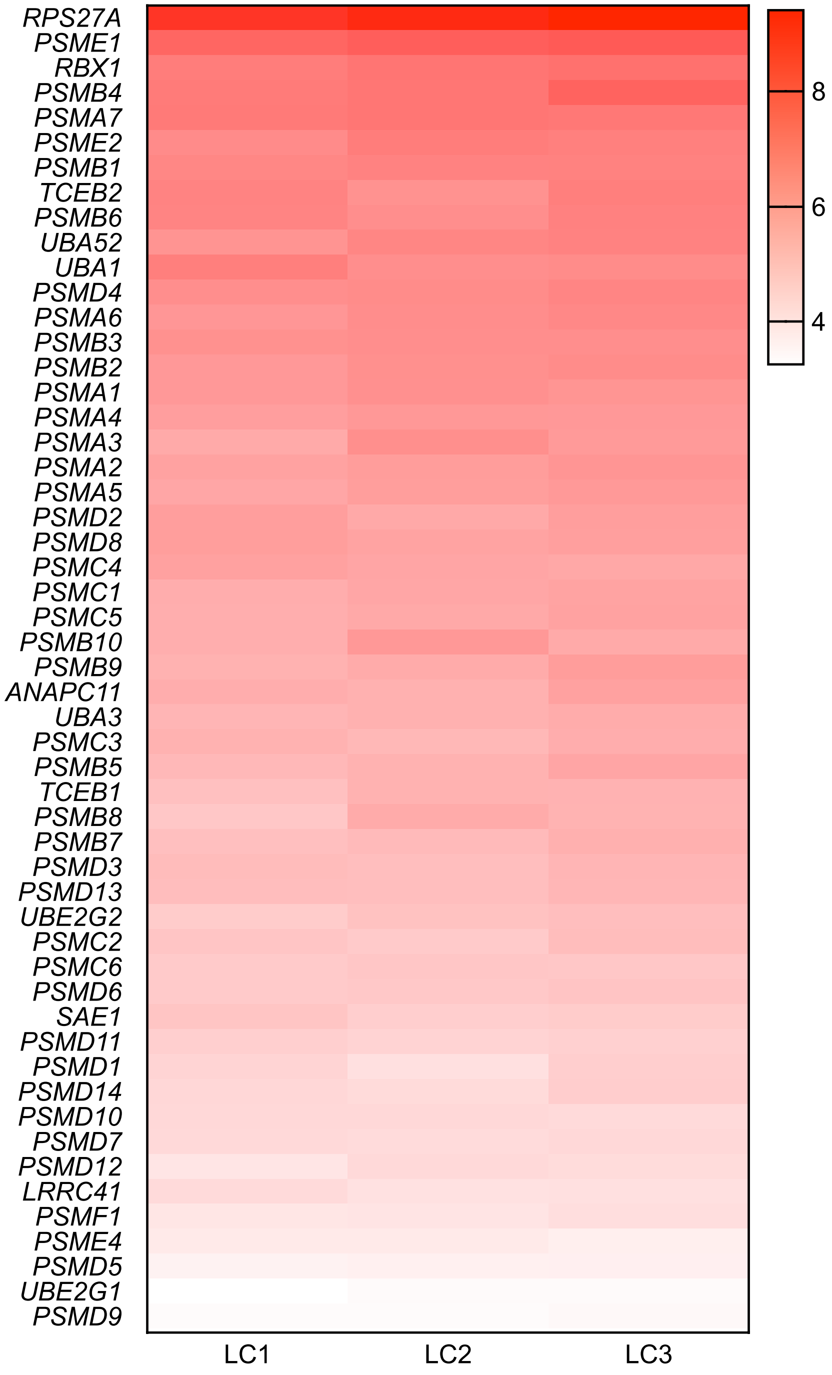

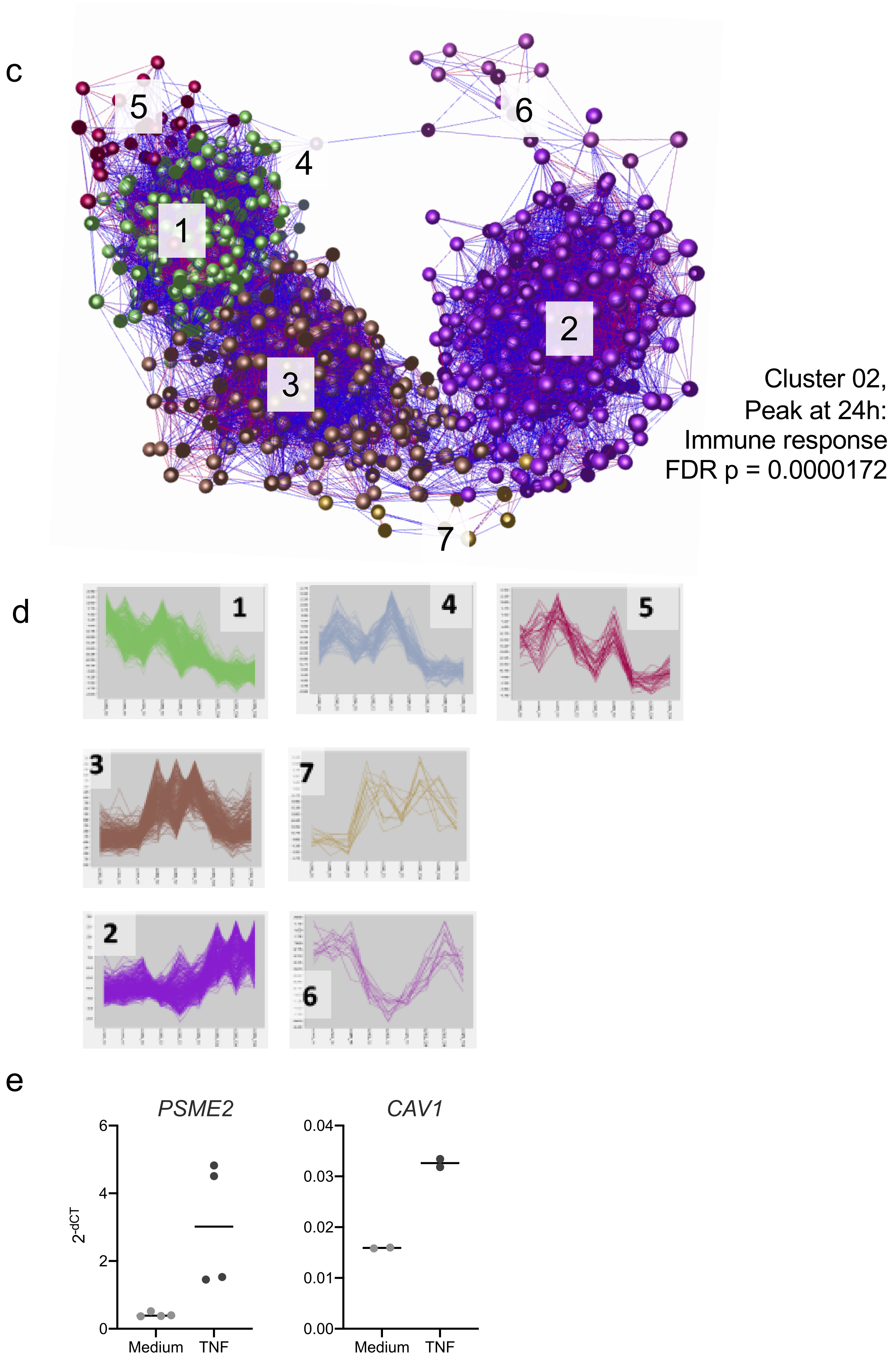
c
5
4
6
1
2
3
7
Cluster 02,
Peak at 24h:
Immune response
FDR p = 0.0000172
d
1
4
5
3
7
2
6
e
PSME2
6
4
2
0
2^{-dCT}
Medium
TNF
CAV1
0.04
0.03
0.02
0.01
0.00
Medium
TNF

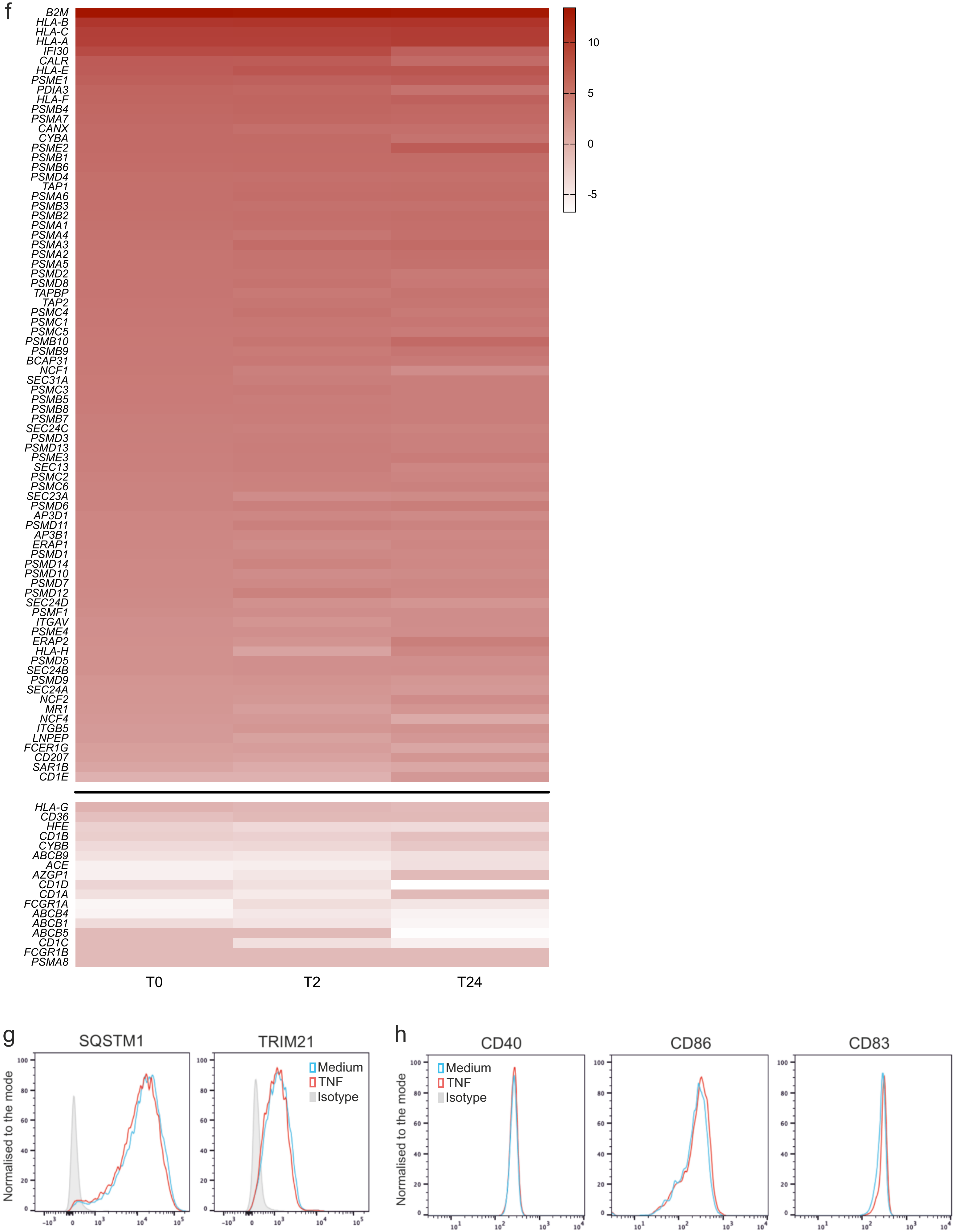
f
B2M
HLA-B
HLA-C
HLA-A
IFI30
CALR
HLA-E
PSME1
PDIA3
HLA-F
PSMB4
PSMA7
CANX
CYBA
PSME2
PSMB1
PSMB6
PSMD4
TAP1
PSMA6
PSMB3
PSMB2
PSMA1
PSMA4
PSMA3
PSMA2
PSMA5
PSMD2
PSMD8
TAPBP
TAP2
PSMC4
PSMC1
PSMC5
PSMB10
PSMB9
BCAP31
NCF1
SEC31A
PSMC3
PSMB5
PSMB8
PSMB7
SEC24C
PSMD3
PSMD13
PSME3
SEC13
PSMC2
PSMC6
SEC23A
PSMD6
AP3D1
PSMD11
AP3B1
ERAP1
PSMD1
PSMD14
PSMD10
PSMD7
PSMD12
SEC24D
PSMF1
ITGAV
PSME4
ERAP2
HLA-H
PSMD5
SEC24B
PSMD9
SEC24A
NCF2
MR1
NCF4
ITGB5
LNPEP
FCER1G
CD207
SAR1B
CD1E
HLA-G
CD36
HFE
CD1B
CYBB
ABCB9
ACE
AZGP1
CD1D
CD1A
FCGR1A
ABCB4
ABCB1
ABCB5
CD1C
FCGR1B
PSMA8
10
5
0
-5
T0
T2
T24
g
SQSTM1
TRIM21
Medium
TNF
Isotype
Normalised to the mode
h
CD40
CD86
CD83
Medium
TNF
Isotype
Normalised to the mode

Supplementary Figure 2 Transcriptional programming of migrated human LCs

a) Overlaps between reported cross-presentation signatures: Reactome database (76 genes), Artyomov et al (311 genes) and genes expressed in migrated LC >10 FPKM.
b) Heatmap of log(2) FPKM gene expression levels for genes involved in antigen processing and presentation in class I, MSigDB, Broad Institute. Source data are provided as a Source Data file
c) Transcript-to-transcript clustering, (BioLayout Express3D, r= 0.80; MCL = 1.7) of 1,156 probesets differentially regulated by TNF. Lines (edges) represent the similarity between transcripts, circles (nodes) represent genes. DGE: 1156, FDR<0.05, |LogFC|>1. 7 main clusters were identified (gene n>8), denoted by different colours. Enrichment in biological processes was done using ToppGene on-line tool significance denoted by FDR (Benjamini-Hochberg) corrected p-value is shown.
d) Expression profiles in 7 main clusters (gene n>8) identified with transcript-to-transcript clustering, (BioLayout Express3D, r= 0.85; MCL = 1.7) of 1,156 probesets differentially regulated by TNF in human migrated LCs. Source data are provided as a Source Data file
e) Gene expression of PSME2 and CAV1 in migrated LC assessed by qPCR (expression normalised to house-keeping gene YWHAZ (2-dCT) before (grey bars) and following stimulation with TNF (black bars) either during or post-migration. N=1-3 independent skin donors, in duplicate. Line denotes median.
f) Heat map of genes included in antigen presentation class I signature from Reactome database. Log (2) FPKM median expression values are shown for each gene in the signature. The black line denotes expression cut-off for detection. RNA-seq n=3 independent donors. Source data are provided as a Source Data file
g) A representative example of intracellular and surface protein expression assessed by flow cytometry in control LCs (red, 24h in medium) LC stimulated 24h with TNF (blue) vs isotype ctrl (grey)

Supplementary Figure 3. scRNA-seq analysis of migrated human LCs

950 single migrated epidermal cells highly enriched in LC were subjected to Drop-seq encapsulation and single cell RNA-sequencing Alignment, read filtering, barcode and UMI counting were performed using kallisto-bustools followed by clustering within the python-based Scanpy.

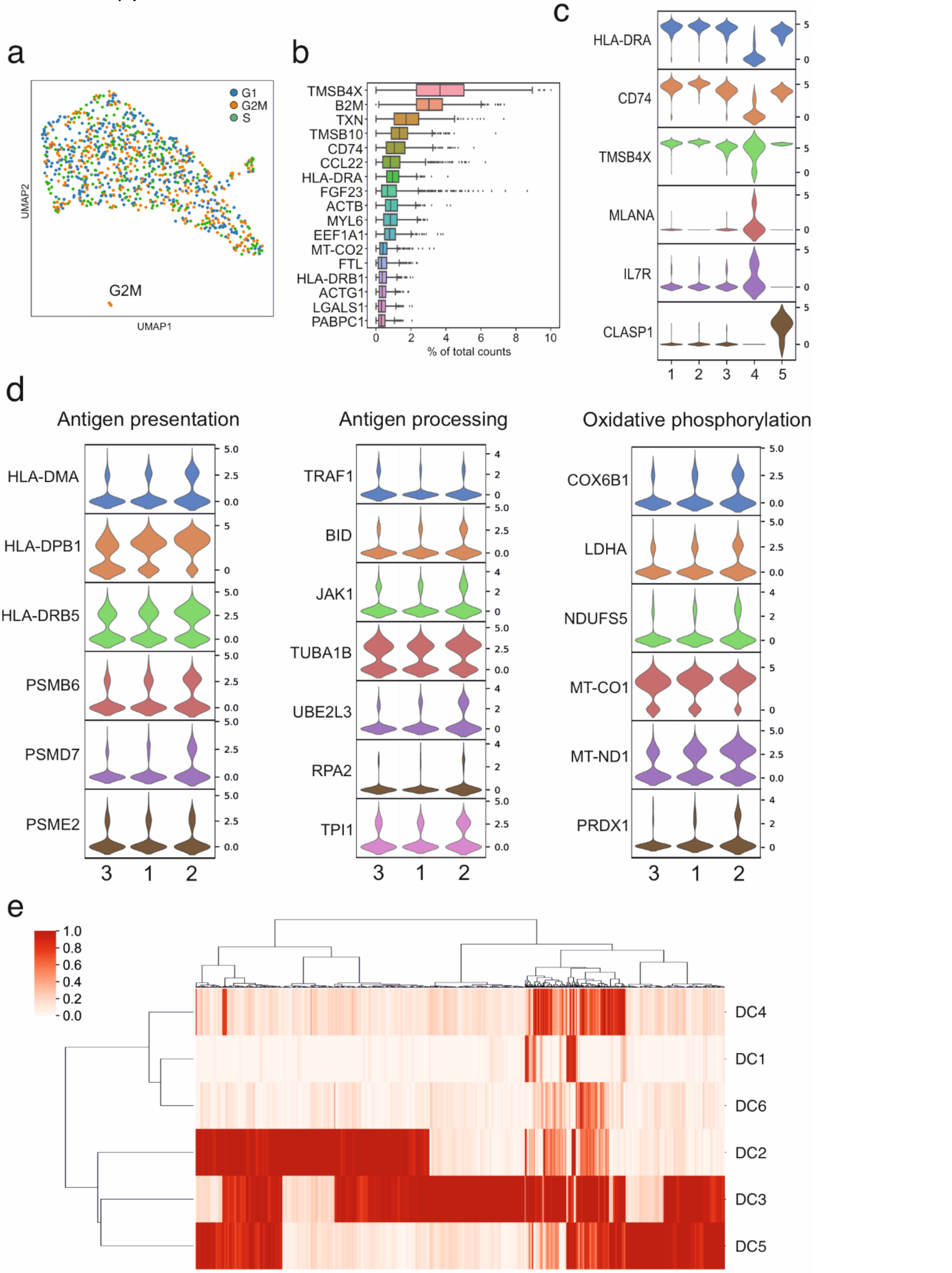

a) UMAP plot of cell cycle analysis indicating that while no cell cycle synchronisation was visible in cluster 1-4, cluster 5 contained exclusively cells in G2S phase (orange).
b) Coverage of gene expression for 20 genes with the highest expression levels detected across all the cells. Percentage of total counts shown, bar indicates median with range for each gene.
c) Violin plots representing expression of cluster defining markers identified with ScanPy analysis, Leiden r=0.2, across cell population.
d) Violin plots representing expression of genes implicated in key biological processes enriched in subclusters of LCs. Genes involved in antigen presentation (left panel), genes involved in antigen processing (middle panel) and genes functioning in oxidative phosphorylation (right panel) are displayed. Each plot shows distribution of CPTT normalised expression values of indicated transcript in a given LC subpopulation.
e) Projection of scRNAseq LC data onto a reference set of newly classified blood dendritic cells was carried out using Gene Set Variation Analysis (GSVA) based on enrichment score. The heatmap shows the GSVA enrichment score for each LC transcriptome.

Supplementary Figure 4. Chromatin landscape of migrated LCs enriches for EICE, AICE and ISRE motifs

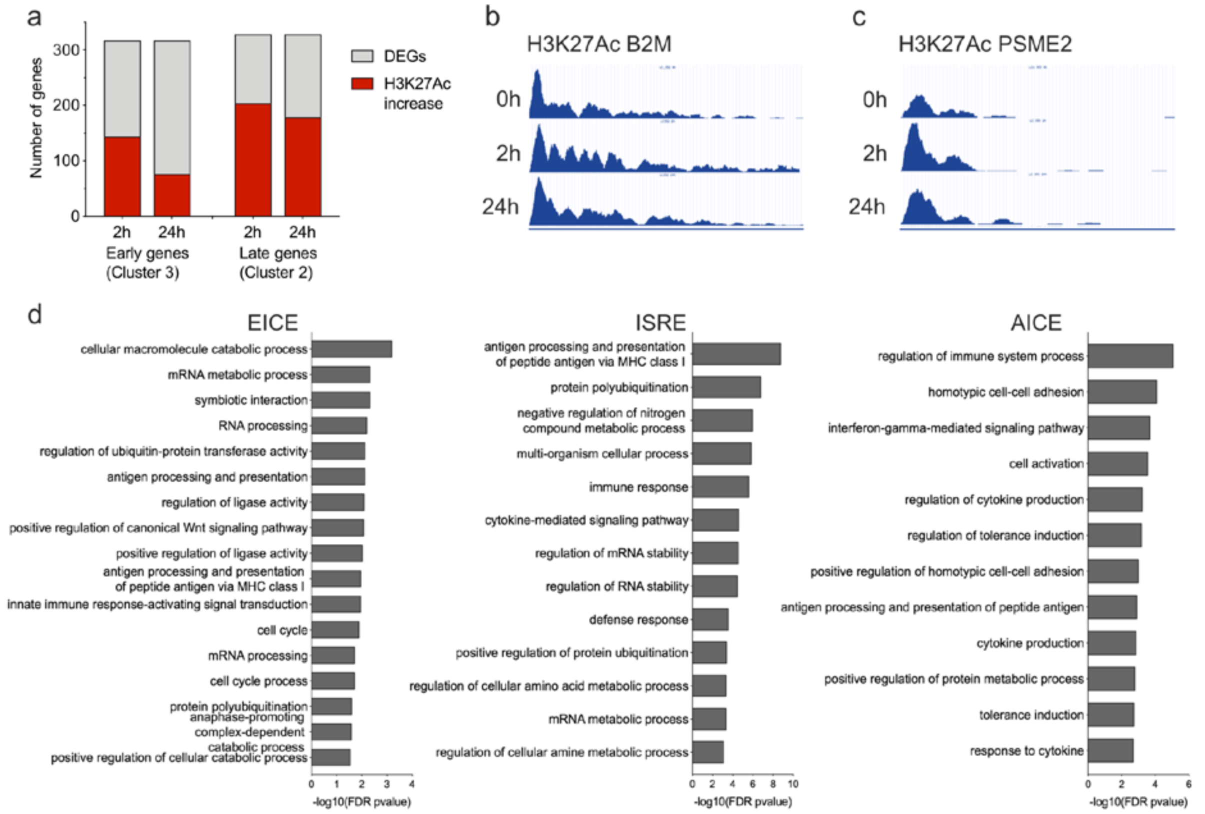

a) Proportion of DEG with increase (M>0) H3K27Ac mark at 2h and 24h in clusters of co-expressed genes up-regulated early (2h, clusters 3) and late (24h, cluster 2) during stimulation with TNF. Changes in H3K27Ac acetylation were calculated using MANorm algorithm (MACS2,[1]) embedded in BioWardrobe tool[2] and genes filtered to include unique common entry across the biological replicates (n=3 independent donors). Genes with detected changes in acetylation were intersected with DEGs identified by EdgeR analysis. Source data are provided as a Source Data file

b) UCSC genome browser tracks of H3K27Ac histone mark for B2M from human LC genomic programme. Acetylated area in the promoter region shown by blue peaks along the gene track.

c) UCSC genome browser tracks of H3K27Ac histone mark for PSME2 from human LC genomic programme. Acetylated area in the promoter region shown by blue peaks along the gene track.

d) Peaks H3K4Me3 and H3K27Ac T0 datasets were scanned for ISRE/AICE/EICE binding motifs. 1193 consensus genes (present in all 3 biological replicates with both chromatin marks) were identified. Biological processes enriched in those genes were detected using ToppGene (FDR corrected p-values for GO categories). Source data are provided as a Source Data file

Supplementary Figure 5. Human LCs upregulate expression of IRF4 upon migration but lack IRF8

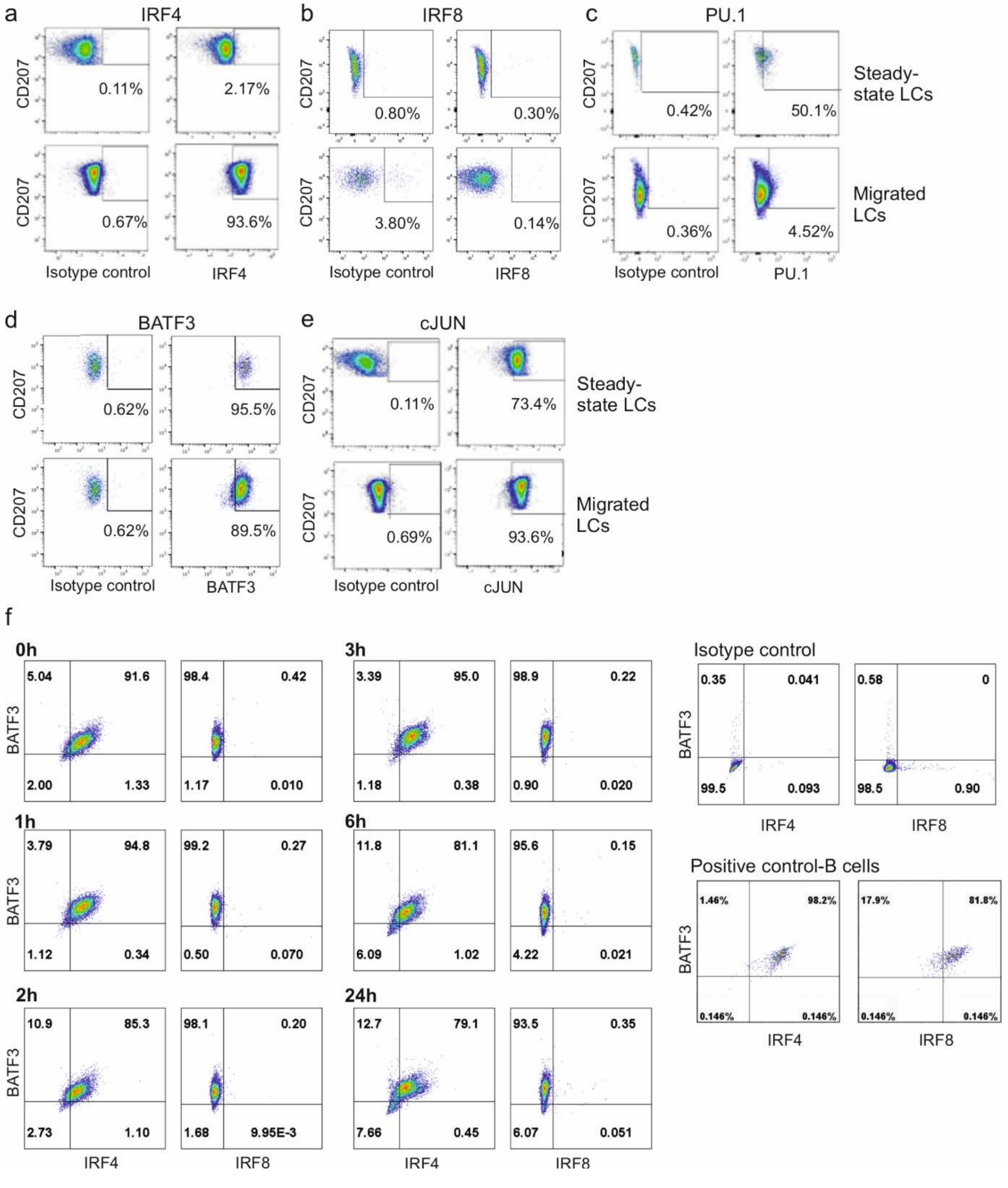

a) Flow cytometry analysis of intranuclear IRF4 protein expression in migrated and steady-state LCs (CD1a/CD207^{high}). Gates set using isotype controls. Data representative of 8 independent donors

b) Flow cytometry analysis of intranuclear IRF8 protein expression in migrated and steady-state LCs (CD1a/CD207^{high}). Gates set using isotype controls. Data representative of 3 independent donors

c) Flow cytometry analysis of intranuclear PU.1 protein expression in migrated and steady-state LCs (CD1a/CD207^{high}). Gates set using isotype controls. Data show an independent experiment (n=1)

d) Flow cytometry analysis of intranuclear BATF3 protein expression in migrated and steady-state LCs (CD1a/CD207^{high}). Gates set using isotype controls. Data representative of 8 independent donors.

e) Flow cytometry analysis of intranuclear cJUN protein expression in migrated and steady-state LCs (CD1a/CD207^{high}). Gates set using isotype controls. Data representative of 2 independent donors.

f) Flow cytometry analysis of intranuclear IRF8 protein expression during a time-course of stimulation with TNF in migrated LCs (CD1a/CD207^{high}). Gates set using isotype controls. Data representative of 2 independent donors.

Supplementary Figure 6. IRF4-mediated transcriptional programming of human LCs

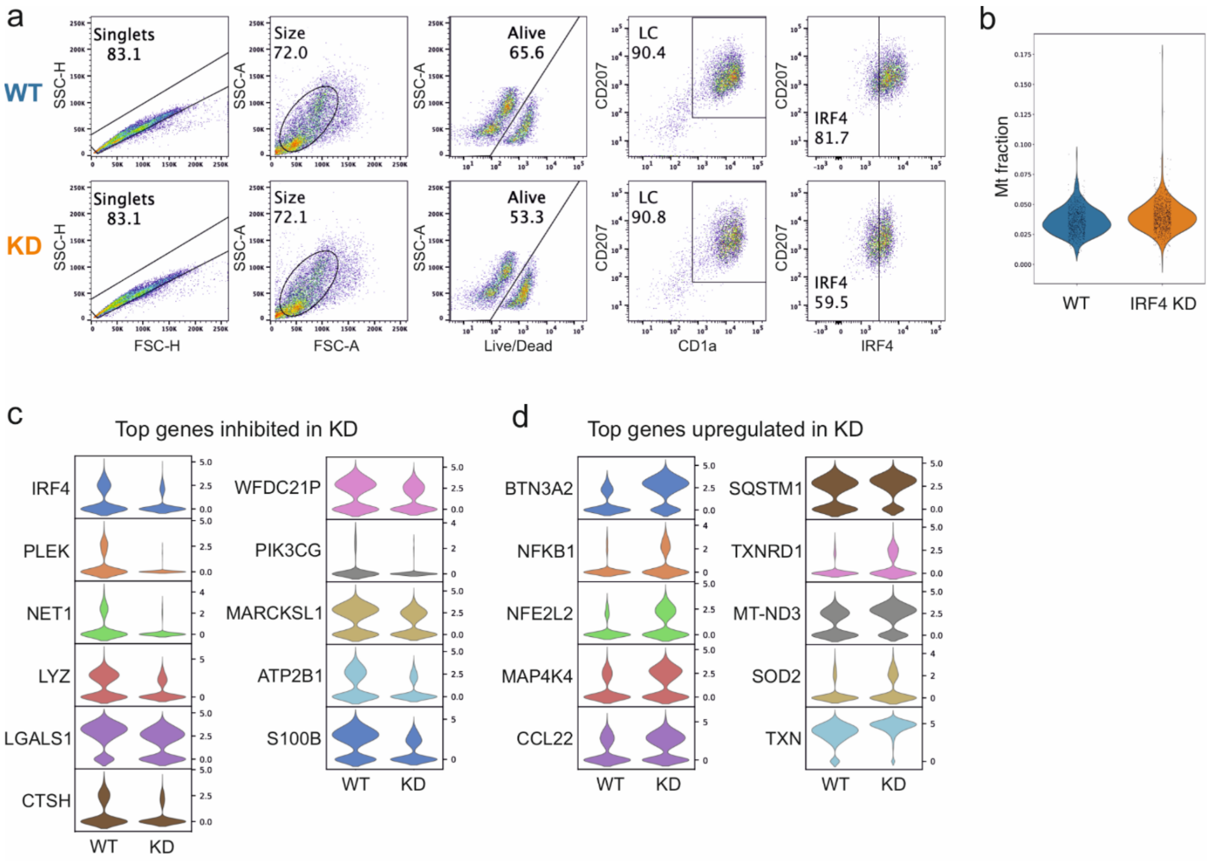

a) Gating strategy for identification of live LCs in migrated cells exposed to CRISPR-Cas9 editing of IRF4 expression using flow cytometry. Gate for IRF4 positive cells was set up based on isotype control fluorescence.

b) Proportion of mitochondrial genes in cells following CRISPR-Cas9 editing in control (WT, orange) and IRF4 edited (KD, blue) single cell transcriptomes

c) Violin plots representing expression of genes up-regulated in IRF4 knock-down (KD) in migrated LCs. Each plot shows distribution of CPTT normalised expression values of indicated transcript in a given LC subpopulation.

d) Violin plots representing expression of genes down-regulated in IRF4 knock-down (KD) in migrated LCs. Each plot shows distribution of CPTT normalised expression values of indicated transcript in a given LC subpopulation.

Supplementary methods

Bulk RNA-seq data analysis

Quality control for FASTQ files with raw sequence data was done using FASTQC tool [FastQC: a quality control tool for high throughput sequence data. Available online at: http://www.bioinformatics.babraham.ac.uk/projects/fastqc] Adapter sequences and low quality reads were trimmed using Trimmomatic [3]. High-quality reads were mapped to the human genome (hg19) using TopHat (version 2.0.9, [4]) and, following the removal of multimapping reads, converted to gene specific read counts for annotated genes using HTSeq-count (version 0.5.4)[5]. Raw counts from RNA-Seq were processed in Bioconductor package EdgeR [6], variance was estimated and size factor normalized using TMM. Genes with minimum 2 reads at minimum 50% samples were included in the downstream analyses. Differentially expressed genes (DEG) we identified applying significance threshold FDR $p<0.05$, $|LogFC|>1$. Normalised reads from DEG were taken for transcript-to-transcript co-expression analysis (BioLayout *Express*[3D],[7]. FPKMs were estimated using Cufflinks package [4]. Gene ontology analysis was done using ToppGene on-line tool [8].

scRNA-seq data analysis

Samples were de-multiplexed with bcl2fastq tool from Illumina. Alignment, read filtering, barcode and UMI counting were performed using kallisto-bustools [9]. All further analyses were run using the python-based Scanpy [10] except where stated otherwise. High quality barcodes were selected based on the overall UMI distribution using emptyDrops [11], filtering criteria was adjusted to match estimated the number of true cells. To remove low quality cells, we filtered cells with a high fraction of counts from mitochondrial genes (20% or more) indicating stressed or dying cells. In addition, genes with expression in less than 5 cells were excluded. Highly variable genes were selected using distribution criteria: min_mean=0.0125, max_mean=6, min_disp=0.6. A single-cell neighbourhood graph was computed on the first principal components that sufficiently explain the variation in the data using 10 nearest neighbours. Uniform Manifold Approximation and Projection (UMAP) was run for visualization. Leiden algorithm [12] was used to identify cell clustering within samples (Leiden r = 0.2, n_pcs=4, n_neighbours =10). Diffmap algorithm was used for pseudotrajectory analysis.

Differentially expressed genes were identified using linear model scDiffExlimma (SingleCellTK package in R[13], FDR corrected p value <0.05 used as a cut-off criteria following scnorm normalisation [14]. Gene enrichment analysis for DEGs was done in ToppGene suit (FDR corrected p value <0.05 cut-off).

To project single cell LC transcriptomes onto known gene signatures from dendritic cell populations we used a set of marker genes from monocytes and DC from Villani et al. as a reference set[15]. Over-representation analysis was performed using the hypergeometric test using GSVA [16]. The results were plotted as a heat map using the Ward method with Euclidean distances.

ChIP-seq data analysis

ChIP-seq data analysis was performed using pipelines implemented in the BioWardrobe suite [2] BAM-formatted hg19 aligned ChIPseq reads were used for peak calling MACS2 (version 2.1.1.[1] was used to estimate fragment size, identify and annotate peaks. Changes in histone modification profiles were assessed with MAnorm algorithm implemented in BioWardrobe. ChiP-seq profiles at a distance -3000bp - +3000bp from TSS were generated using ChIPseeker package in R. Common regions with histone modification were identified using findoverlaps function, DiffBind package (R environment)

Transcription factor binding site motif enrichment analysis

We performed TF motif enrichment analysis on ChIP-seq peak sets using the HOMER software package [17]. HOMER calculates the statistical enrichment in a set of input DNA sequences (here, ChIP-seq peak sequences) for a large set of position weight matrix (PWM) TF binding models. Subsequently, putative binding sites for enriched motifs (EICE, AICE, and ISRE) were identified in the H3K27ac T0 dataset using custom scripts encoding the standard log likelihood PWM scoring function [18]. DNA sequences scoring at least 70% of the best possible log-likelihood score were recorded as putative binding sites.

Intersection of ChIP-seq peaks with public genomics datasets

To identify transcription factor binding events and other epigenetic marks that overlap with our ChIP-seq data, we used our Regulatory Element Locus Intersector (RELI) computational

method[19], which overlaps a set of genomic locations (e.g., peaks from a ChIP-seq experiment) with a large collection of functional genomics datasets. We created a library of ~5,000 datasets by compiling data (ChIP-seq for TFs and histone marks, DNase-seq, ATAC-seq, etc.) from a variety of sources, including ENCODE [20], Cistrome [21], PAZAR [22], Re-Map [23], and Roadmap Epigenomics [24]. As input, RELI takes a set of genomic loci (e.g., H3K27ac ChIP-seq peaks at T0). These loci are systematically intersected with each functional genomics dataset, and the number of input regions overlapping each dataset by at least one base are counted. Next, a p-value describing the significance of this overlap is estimated using a simulation-based procedure. To this end, a 'negative set' is created for comparison to the input set, which is created by compiling all regions of open chromatin in the genome (i.e., the union of all available human DNAse-seq, ATAC-seq, and FAIRE-seq datasets). A distribution of expected overlap values is then created from 2,000 iterations of randomly sampling from the negative set, each time choosing a set of negative examples that match the input set in terms of the total number of genomic loci and the length of each locus. The distribution of the expected overlap values is used to generate a Z-score and corresponding p-value estimating the significance of the observed number of input regions that overlap each data set. Collectively, this procedure controls for the count and sizes of the input loci, and the count and sizes of each individual dataset in the library.

1 Zhang, Y. *et al.* Model-based analysis of ChIP-Seq (MACS). *Genome biology* **9**, R137, doi:10.1186/gb-2008-9-9-r137 (2008).

2 Kartashov, A. V. & Barski, A. BioWardrobe: an integrated platform for analysis of epigenomics and transcriptomics data. *Genome biology* **16**, 158, doi:10.1186/s13059-015-0720-3 (2015).

3 Bolger, A. M., Lohse, M. & Usadel, B. Trimmomatic: a flexible trimmer for Illumina sequence data. *Bioinformatics (Oxford, England)* **30**, 2114-2120, doi:10.1093/bioinformatics/btu170 (2014).

4 Trapnell, C. *et al.* Differential gene and transcript expression analysis of RNA-seq experiments with TopHat and Cufflinks. *Nat Protoc* **7**, 562-578, doi:10.1038/nprot.2012.016 (2012).

5 Anders, S., Pyl, P. T. & Huber, W. HTSeq--a Python framework to work with high-throughput sequencing data. *Bioinformatics (Oxford, England)* **31**, 166-169, doi:10.1093/bioinformatics/btu638 (2015).

6 Robinson, M. D., McCarthy, D. J. & Smyth, G. K. edgeR: a Bioconductor package for differential expression analysis of digital gene expression data. *Bioinformatics (Oxford, England)* **26**, 139-140, doi:10.1093/bioinformatics/btp616 (2010).

7 Theocharidis, A., van Dongen, S., Enright, A. J. & Freeman, T. C. Network visualization and analysis of gene expression data using BioLayout Express(3D). *Nat Protoc* **4**, 1535-1550, doi:10.1038/nprot.2009.177 (2009).
8 Chen, J., Bardes, E. E., Aronow, B. J. & Jegga, A. G. ToppGene Suite for gene list enrichment analysis and candidate gene prioritization. *Nucleic acids research* **37**, W305-311, doi:10.1093/nar/gkp427 (2009).
9 Bray, N. L., Pimentel, H., Melsted, P. & Pachter, L. Near-optimal probabilistic RNA-seq quantification. *Nature biotechnology* **34**, 525, doi:10.1038/nbt.3519
https://www.nature.com/articles/nbt.3519#supplementary-information (2016).
10 Wolf, F. A., Angerer, P. & Theis, F. J. SCANPY: large-scale single-cell gene expression data analysis. *Genome biology* **19**, 15, doi:10.1186/s13059-017-1382-0 (2018).
11 Lun, A. T. L. *et al.* EmptyDrops: distinguishing cells from empty droplets in droplet-based single-cell RNA sequencing data. *Genome biology* **20**, 63, doi:10.1186/s13059-019-1662-y (2019).
12 Traag, V. A., Waltman, L. & van Eck, N. J. From Louvain to Leiden: guaranteeing well-connected communities. *Scientific reports* **9**, 5233, doi:10.1038/s41598-019-41695-z (2019).
13 singleCellTK: Interactive Analysis of Single Cell RNA-Seq Data. R package version 1.4.2, (GitHub, 2019).
14 Bacher, R. *et al.* SCnorm: robust normalization of single-cell RNA-seq data. *Nature methods* **14**, 584-586, doi:10.1038/nmeth.4263 (2017).
15 Villani, A. C. *et al.* Single-cell RNA-seq reveals new types of human blood dendritic cells, monocytes, and progenitors. *Science (New York, N.Y.)* **356**, doi:10.1126/science.aah4573 (2017).
16 Hänzelmann, S., Castelo, R. & Guinney, J. GSVA: gene set variation analysis for microarray and RNA-Seq data. *BMC bioinformatics* **14**, 7, doi:10.1186/1471-2105-14-7 (2013).
17 Heinz, S. *et al.* Simple combinations of lineage-determining transcription factors prime cis-regulatory elements required for macrophage and B cell identities. *Molecular cell* **38**, 576-589, doi:10.1016/j.molcel.2010.05.004 (2010).
18 Stormo, G. D. DNA binding sites: representation and discovery. *Bioinformatics (Oxford, England)* **16**, 16-23, doi:10.1093/bioinformatics/16.1.16 (2000).
19 Harley, J. B. *et al.* Transcription factors operate across disease loci, with EBNA2 implicated in autoimmunity. *Nature genetics* **50**, 699-707, doi:10.1038/s41588-018-0102-3 (2018).
20 consortium, E. p. An integrated encyclopedia of DNA elements in the human genome. *Nature* **489**, 57-74, doi:10.1038/nature11247 (2012).
21 Liu, T. *et al.* Cistrome: an integrative platform for transcriptional regulation studies. *Genome biology* **12**, R83, doi:10.1186/gb-2011-12-8-r83 (2011).
22 Portales-Casamar, E. *et al.* The PAZAR database of gene regulatory information coupled to the ORCA toolkit for the study of regulatory sequences. *Nucleic acids research* **37**, D54-60, doi:10.1093/nar/gkn783 (2009).
23 Griffon, A. *et al.* Integrative analysis of public ChIP-seq experiments reveals a complex multi-cell regulatory landscape. *Nucleic acids research* **43**, e27, doi:10.1093/nar/gku1280 (2015).
24 Chadwick, L. H. The NIH Roadmap Epigenomics Program data resource. *Epigenomics* **4**, 317-324, doi:10.2217/epi.12.18 (2012).
