# Supplementary material for: Genomic programming of IRF4-expressing human Langerhans cells
Source: Nat Commun. 2020 Jan 16;11:313. doi: 10.1038/s41467-019-14125-x (PMC6965086; doi:10.1038/s41467-019-14125-x)
Supplement: Supplementary file 3 — Reporting Summary [file 41467_2019_14125_MOESM3_ESM.pdf]

nature research

Corresponding author(s): m.e.polak@soton.ac.uk

Last updated by author(s): 16/12/19

Reporting Summary

Nature Research wishes to improve the reproducibility of the work that we publish. This form provides structure for consistency and transparency in reporting. For further information on Nature Research policies, seeAuthors & Referees and theEditorial Policy Checklist .

Statistics

For all statistical analyses, confirm that the following items are present in the figure legend, table legend, main text, or Methods section.

n/a	Confirmed	
☐	☒	The exact sample size (*n*) for each experimental group/condition, given as a discrete number and unit of measurement
☐	☒	A statement on whether measurements were taken from distinct samples or whether the same sample was measured repeatedly
☐	☒	The statistical test(s) used AND whether they are one- or two-sided<br>*Only common tests should be described solely by name; describe more complex techniques in the Methods section.*
☐	☒	A description of all covariates tested
☐	☒	A description of any assumptions or corrections, such as tests of normality and adjustment for multiple comparisons
☐	☒	A full description of the statistical parameters including central tendency (e.g. means) or other basic estimates (e.g. regression coefficient) AND variation (e.g. standard deviation) or associated estimates of uncertainty (e.g. confidence intervals)
☐	☒	For null hypothesis testing, the test statistic (e.g. *F*, *t*, *r*) with confidence intervals, effect sizes, degrees of freedom and *P* value noted<br>*Give P values as exact values whenever suitable.*
☐	☒	For Bayesian analysis, information on the choice of priors and Markov chain Monte Carlo settings
☐	☒	For hierarchical and complex designs, identification of the appropriate level for tests and full reporting of outcomes
☒	☐	Estimates of effect sizes (e.g. Cohen's *d*, Pearson's *r*), indicating how they were calculated

Our web collection on statistics for biologists contains articles on many of the points above.

Software and code

Policy information about availability of computer code

Data collection	N/A
Data analysis	Bulk RNA analysis: FASTQC tool [FastQC: a quality control tool for high throughput sequence data. Available online at: http://www.bioinformatics.babraham.ac.uk/projects/fastqc] Adapter sequences and low quality reads were trimmed using Trimmomatic {Bolger, 2014 #3146}. High-quality reads were mapped to the human genome (hg19) using TopHat (version 2.0.9, {Trapnell, 2012 #3150}) and, following the removal of multimapping reads, converted to gene specific read counts for annotated genes using HTSeq-count (version 0.5.4) {Anders, 2015 #3145}. Raw counts from RNA-Seq were processed in Bioconductor package EdgeR {Robinson, 2010 #3152}, variance was estimated and size factor normalized using TMM. FPKMs were estimated using Cufflinks package {Trapnell, 2012 #3150}. Gene ontology analysis was done using ToppGene on-line tool {Chen, 2009 #2503}.<br>single cell data analysis: Alignment, read filtering, barcode and UMI counting were performed using kallisto-bustools {Bray, 2016 #2896}. All further analyses were run using the python-based Scanpy {Wolf, 2018 #3135}. Leiden algorithm {Traag, 2019 #2898} was used to identify cell clustering within samples. Leiden algorithm {Traag, 2019 #2898} was used to identify cell clustering within samples (Leiden r = 0.2, n_pcs=4, n_neighbours =10). Diffmap algorithm was used for pseudotrajectory analysis. Differentially expressed genes were identified using linear model scDiffExlimma (SingleCellTK package V1.4.1)<br>ChIP-seq data analysis: To identify transcription factor binding events and other epigenetic marks that overlap with our ChIP-seq data, we used our Regulatory Element Locus Intersector (RELI) computational method {Harley, 2018 #3139}

For manuscripts utilizing custom algorithms or software that are central to the research but not yet described in published literature, software must be made available to editors/reviewers. We strongly encourage code deposition in a community repository (e.g. GitHub). See the Nature Research guidelines for submitting code & software for further information.

Data

Policy information about availability of data

All manuscripts must include a data availability statement. This statement should provide the following information, where applicable:

- Accession codes, unique identifiers, or web links for publicly available datasets
- A list of figures that have associated raw data
- A description of any restrictions on data availability

Sequencing data for RNA-seq, scRNA-seq and ChiP-seq is stored in Gene Expression Omnibus database, submission number GSE120386.

Field-specific reporting

Please select the one below that is the best fit for your research. If you are not sure, read the appropriate sections before making your selection.

- [x] Life sciences
- [ ] Behavioural & social sciences
- [ ] Ecological, evolutionary & environmental sciences

For a reference copy of the document with all sections, see nature.com/documents/nr-reporting-summary-flat.pdf

Life sciences study design

All studies must disclose on these points even when the disclosure is negative.

Sample size	This is a pilot study. N=3 biological replicates were used for bulk RNA-seq for each time point. n=1000 single cell transcriptomes were used for scRNA-sequencing
Data exclusions	Data was subject to quality checks using FASTQC for bulk RNA seq and barcode and cell quality for scRNA seq. All samples passed the quality criteria
Replication	The data was assessed in 3 biological donors
Randomization	no randomisation was used
Blinding	no blinding was in place

Reporting for specific materials, systems and methods

We require information from authors about some types of materials, experimental systems and methods used in many studies. Here, indicate whether each material, system or method listed is relevant to your study. If you are not sure if a list item applies to your research, read the appropriate section before selecting a response.

Materials & experimental systems

n/a	Involved in the study	
[ ]	[x]	Antibodies
[x]	[ ]	Eukaryotic cell lines
[x]	[ ]	Palaeontology
[x]	[ ]	Animals and other organisms
[ ]	[x]	Human research participants
[x]	[ ]	Clinical data

Methods

n/a	Involved in the study	
[ ]	[x]	ChIP-seq
[ ]	[x]	Flow cytometry
[x]	[ ]	MRI-based neuroimaging

Antibodies

Antibodies used	of CD207, CD1a, HLA-DR (mouse monoclonal antibodies, CD1a, CD207:Miltenyi Biotech, UK and HLA-DR: BD Biosciences, UK). IRF4, IRF8, BATF3, PU.1, cJUN, (IRF4:rat monoclonal, eBiosciences, UK, mouse monoclonal: IRF8, eBiosciences, BATF, R&D Systems, JUN Millimark, UK, PU.1 Biolegend, UK). IRF1 staining was done using rabbit polyclonal anti-human IRF1 antibody (Abcam, UK)
Validation	All antibodies were used at pre-titrated concentration. The specificity was detailed in the antibody datasheet. Appropriate isotype controls were used to assess the baseline fluorescence.

Human research participants

Policy information about studies involving human research participants

Population characteristics	Recruited donors were adult, male and female. Samples were anonymised, and no personal data was collected
Recruitment	Skin specimens were acquired from healthy individuals undergoing plastic surgery (dicarded tissue). Subjects were recruited to the study by a clinician performing the procedure.<br>Blood samples were collected from healthy volunteers recruited to the study from University staff and students.
Ethics oversight	Skin specimens and blood samples were acquired from healthy individuals after obtaining informed written consent with approval by the Southampton and South West Hampshire Research Ethics Committee in adherence to Helsinki Guidelines [ethical approvals: 07/Q1704/59, NRES 07 Q1704 46].

Note that full information on the approval of the study protocol must also be provided in the manuscript.

ChIP-seq

Data deposition

- [x] Confirm that both raw and final processed data have been deposited in a public database such as GEO.
- [x] Confirm that you have deposited or provided access to graph files (e.g. BED files) for the called peaks.

Data access links
May remain private before publication.

ChiP-seq is stored in Gene Expression Omnibus database, submission number GSE120386.

Files in database submission

Raw datafiles
LC1_T0_H3K4me3.fastq
LC1_2h_H3K4me3.fastq
LC1_24h_H3K4me3.fastq
LC2_T0_H3K4me3.fastq
LC2_2h_H3K4me3.fastq
LC2_24h_H3K4me3.fastq
LC3_T0_H3K4me3.fastq
LC3_2h_H3K4me3.fastq
LC3_24h_H3K4me3.fastq
LC1_T0_H3K27ac.fastq
LC1_2h_H3K27ac.fastq
LC1_24h_H3K27ac.fastq
LC2_T0_H3K27ac.fastq
LC2_2h_H3K27ac.fastq
LC2_24h_H3K27ac.fastq
LC3_T0_H3K27ac.fastq
LC3_2h_H3K27ac.fastq
LC3_24h_H3K27ac.fastq
LC1_T0_read1.fastq.gz
LC1_T2_read1.fastq.gz
LC1_T24_read1.fastq.gz
LC2_T0_read1.fastq.gz
LC2_T2_read1.fastq.gz
LC2_T24_read1.fastq.gz
LC3_T0_read1.fastq.gz
LC3_T2_read1.fastq.gz
LC3_T24_read1.fastq.gz
LC4_150_R1.fastq.gz
LC1_T0_read2.fastq.gz
LC1_T2_read2.fastq.gz
LC1_T24_read2.fastq.gz
LC2_T0_read2.fastq.gz
LC2_T2_read2.fastq.gz
LC2_T24_read2.fastq.gz
LC3_T0_read2.fastq.gz
LC3_T2_read2.fastq.gz
LC3_T24_read2.fastq.gz
LC4_150_R2.fastq.gz
S0_R1_001.fastq.gz
CTR_S1_R1_001.fastq.gz
IRF4_CRISPR_S2_R1_001.fastq.gz

LC1_T0.bam
LC1_T2.bam
LC1_T24.bam
LC2_T0.bam
LC2_T2.bam
LC2_T24.bam
LC3_T0.bam
LC3_T2.bam
LC3_T24.bam
Processed datafiles
LC1_0h_me3.csv
LC1_2h_me3.csv
LC1_24h_me3.csv
LC2_0h_me3.csv
LC2_2h_me3.csv
LC2_24h_me3.csv
LC3_0h_me3.csv
LC3_2h_me3.csv
LC3_24h_me3.csv
LC1_0h_ac.csv
LC1_2h_ac.csv
LC1_24h_ac.csv
LC2_0h_ac.csv
LC2_2h_ac.csv
LC2_24h_ac.csv
LC3_0h_ac.csv
LC3_2h_ac.csv
LC3_24h_ac.csv
LC1_T0.txt
LC1_T2.txt
LC1_T24.txt
LC2_T0.txt
LC2_T2.txt
LC2_T24.txt
LC3_T0.txt
LC3_T2.txt
LC3_T24.txt
LC4_150_out_gene_exon_tagged.dge.txt
LC_5_T0_1000
LC_6_CRISPR_CTRL_1000
LC_6_CRISPR_IRF4KD_1000

Genome browser session (e.g. UCSC)

N/A

Methodology

Replicates

n=3 biological replicates for RNAseq bulk and ChiP-seq experiment. n=1000 single cells for each single cell RNA-seq experiment

Sequencing depth

Bulk RNA-seq 75,000,000 reads per sample, paired end. Chip-seq 35,000,000 reads per sample, single cell RNA seq 100,000 reads per cell

Antibodies

rabbit polyclonal anti-H3K27ac antibody (Diagenode Inc.), a rabbit monoclonal anti-H3K4me3 antibody (17-614, Millipore),

Peak calling parameters

BAM-formatted hg19 aligned ChIPseq reads were used for peak calling MACS2 (version 2.1.1) within a tool BioWardrobe. Changes in histone modification profiles were assessed with MAnorm algorithm implemented in BioWardrobe. ChiP-seq profiles at a distance -3000bp - +3000bp from TSS were generated using ChIPseeker package in R.

Data quality

FDR corrected p values were used, as well as cosisten calling across the biological replicates. 13,402 H3K4Me3 and 11,665 H3K4Ac peaks were detected above the 5% FDR p value threshold

Software

ChIP-seq data analysis was performed using pipelines implemented in the BioWardrobe suite {Kartashov, 2015 #3143} BAM-formatted hg19 aligned ChIPseq reads were used for peak calling MACS2 (version 2.1.1. {Zhang, 2008 #3154} was used to estimate fragment size, identify and annotate peaks. Changes in histone modification profiles were assessed with MAnorm algorithm implemented in BioWardrobe. ChiP-seq profiles at a distance -3000bp - +3000bp from TSS were generated using ChIPseeker package in R. Common regions with histone modification were identified using findoverlaps function, DiffBind package (R environment.

Flow Cytometry

Plots

Confirm that:

- [x] The axis labels state the marker and fluorochrome used (e.g. CD4-FITC).
- [x] The axis scales are clearly visible. Include numbers along axes only for bottom left plot of group (a 'group' is an analysis of identical markers).
- [x] All plots are contour plots with outliers or pseudocolor plots.
- [x] A numerical value for number of cells or percentage (with statistics) is provided.

Methodology

Sample preparation	To facilitate the analysis of the genomic programming of primary human LCs, we utilized established protocols for isolating highly pure populations of viable and functional LCs from the epidermis, {Polak, 2014 #2259;Polak, 2012 #1027}. Split skin was obtained using graft knife and subjected to dispase (2U/ml, Gibco, UK, 20h, +4℃) digestion of epidermal sheets. Migrated LCs were harvested after 48 hours culture of epidermal fragments in full culture media (RPMI, Gibco, UK, 5%FBS, Invitrogen, UK, 100 IU/ml penicillin and 100 mg/ml streptomycin, Sigma, UK). Low density cells were enriched using density gradient centrifugation (Optiprep 1:4.2, Axis Shield, Norway {Polak, 2012 #2083} and purified with CD1a+ magnetic beads according to manufacturer's protocol (Milenyi Biotec, UK). Migrated LCs were processed for RNA-seq and ChIP-seq experiment or immediately cryopreserved in 90% FBS (Gibco, UK), 10% DMSO (Sigma, UK). For genomic and transcriptomic analyses of activated LCs fresh migrated LCs from 3 donors were stimulated with TNF-α (25 ng/ml, Miltenyi Biotec, UK) for 2, and 24 hours (RNA-seq: 3 x 105 cells/donor/time point, ChIP-seq: 1.5-2 x 106 cells/donor/time point, paired samples from the same donor for RNA-seq and ChIP-seq). Steady-state LCs were enzymatically digested from the epidermal sheets using LiberaseTM TM research grade (Roche, UK, 2h at 37℃).
Instrument	Aria I, BD
Software	Flow Jo
Cell population abundance	Langerhas cell constituted >95% cells post sorting
Gating strategy	Singlets - live cells-CD207/CD1a double positive cells

- [x] Tick this box to confirm that a figure exemplifying the gating strategy is provided in the Supplementary Information.
