# Supplementary material for: Genomic programming of IRF4-expressing human Langerhans cells
Source: Nat Commun. 2020 Jan 16;11:313. doi: 10.1038/s41467-019-14125-x (PMC6965086; doi:10.1038/s41467-019-14125-x)
Supplement: Supplementary file 4 — Description of Additional Supplementary Files [file 41467_2019_14125_MOESM4_ESM.pdf]

Description of Additional Supplementary Files

File Name: Supplementary Data 1

Description: Cross-presentation molecular signatures from publically available datasets (MSigDB, Reactome, Artyomov et al 2015) and their overlaps with genes expressed in human migrated Langerhans cells

File Name: Supplementary Data 2

Description: Analysis of gene ontology encoded in clusters from bulk RNA-seq analysis, human migrated LCs. Transcript-to-transcript clustering, (BioLayout Express3D, r= 0.80; MCL = 1.7) of 1,156 probesets differentially regulated by TNF.

File Name: Supplementary Data 3

Description: Cluster markers for single cell transcriptome analysis in migrated LCs. 950 single migrated epidermal cells highly enriched in LC were subjected to Drop-seq encapsulation and single cell RNA-sequencing Alignment, read filtering, barcode and UMI counting were performed using kallisto-bustools followed by clustering within the python-based Scanpy, Leiden r=0.2

File Name: Supplementary Data 4

Description: LCMe3_T0_analysis. H3K4Me3 peaks identified in human migrated LCs. MACS2 (version 2.1.1)

File Name: Supplementary Data 5

Description: LCAc27_T0_analysis. H3K27Ac peaks identified in human migrated LCs. MACS2 (version 2.1.1)

File Name: Supplementary Data 6

Description: Intersection of ChIP-seq peaks with public genomics datasets. Overlaps between H3K4Me3 and H3K27Ac peaks in human Langerhans cells and a library of ~5,000 datasets (ChIP-seq for TFs and histone marks, DNase-seq, ATAC-seq, etc.) from a variety of sources, including ENCODE, Cistrome, PAZAR, Re-Map, and Roadmap Epigenomics identified using Regulatory Element Locus Intersector (RELI) computational method.

File Name: Supplementary Data 7

Descriptioion: TF motif enrichment analysis on ChIP-seq peak sets from human migrated Langerhans cells using the HOMER software package

File Name: Supplementary Data 8

Description: Genes and processes high in control migrated Langerhans cells. Differentially expressed genes were identified using linear model scDiffExlimma (SingleCellTK package in R, FDR corrected p value <0.05 used as a cut-off criteria following scnorm normalisation. Gene enrichment analysis for DEGs was done in ToppGene suit (FDR corrected p value <0.05 cut-off).

File Name: Supplementary Data 9

Description: Genes and processes high in IRF4 KD migrated Langerhans cells. Differentially expressed genes were identified using linear model scDiffExlimma (SingleCellTK package in R, FDR corrected p value <0.05 used as a cut-off criteria following scnorm normalisation. Gene enrichment analysis for DEGs was done in ToppGene suit (FDR corrected p value <0.05 cut-off).
